# Temperature Dominates Light in Regulating Lycopene During a Critical Period in Postharvest Tomato Fruit

**DOI:** 10.3390/ijms27114690

**Published:** 2026-05-22

**Authors:** Jinyan Chen, Chenyang He, Qu Luo, Yujuan Zhong, Yingchao Xu, Jiayu Luo, Huaiyuan Li, Xuelian Zhang

**Affiliations:** 1 College of Life Sciences, South China Agricultural University, Guangzhou 510642, China; cjy505@stu.scau.edu.cn (J.C.); hcy202334301065@stu.jnu.edu.cn (C.H.); luoqv0122@163.com (Q.L.); 20243184048@stu.scau.edu.cn (J.L.); 2 Guangdong Key Laboratory for New Technology Research of Vegetables, Vegetable Research Institute, Guangdong Academy of Agricultural Sciences, Guangzhou 510640, China; zhongyujuan@gdaas.cn (Y.Z.); xuyingchao@gdaas.cn (Y.X.); 3 National Demonstration Center for Experimental Essentials of Plant Biology Education (SCAU)/Basic Experiment and Practice Training Center, South China Agricultural University, Guangzhou 510642, China; leehy@scau.edu.cn

**Keywords:** tomato fruit, chlorophyll, lycopene, temperature, high light

## Abstract

Fruit coloration is a key determinant of tomato quality, yet how light and temperature interact to regulate pigmentation during ripening remains unclear. Using a semi-in-fruit experimental system, we demonstrate that while high light accelerates chlorophyll degradation and lycopene accumulation at 25 °C, supra-optimal temperature (40 °C) completely abolishes lycopene biosynthesis irrespective of light conditions, primarily through transcriptional suppression of *SlPSY1* and *SlGGPS2*. Elevated postharvest temperatures (≥30 °C) not only change the carotenoid composition but also reduce the antioxidant capacity and vitamin C content in fruit. Temperature-switch experiments revealed a critical developmental window, days 2–4 after ethylene treatment, during which temperature exerts dominant control over carotenoid metabolism. Exposure to high temperature within this window irreversibly shifts pigment accumulation from lycopene to yellow/orange carotenoids. These findings identify a temporally precise regulatory nexus integrating environmental signals with the ripening program, offering a framework for targeted temperature management to optimize tomato color and nutritional quality.

## 1. Introduction

Tomato (*Solanum lycopersicum*) is a globally significant vegetable crop, valued not only for its widespread cultivation but also for its nutritional composition, being a rich source of vitamins, antioxidants, and carotenoids [1]. Among quality attributes, fruit coloration serves as a primary visual indicator of ripeness and a key determinant of consumer preference and marketability. This color transition, from green to red, is orchestrated by the coordinated degradation of chlorophylls and the de novo biosynthesis and accumulation of carotenoids, most notably the red pigment lycopene [2].

All the genes in the chlorophyll degradation and carotenoid biosynthetic pathway have been isolated and well characterized [3,4,5,6]. Chlorophyll degradation is initiated by STAY-GREEN1 (*SGR1*) [7], leading to dephytylation by pheophytinase (*PPH*) [8,9,10] and subsequent steps catalyzed by pheophorbide a oxygenase (*PaO*) [11] and red chlorophyll catabolite reductase (*RCCR*) [12]. Concurrently, carotenoid biosynthesis commences with the condensation of geranylgeranyl diphosphate (*GGPP*) by phytoene synthase (*PSY1*) [13,14], the rate-limiting step, followed by a series of desaturation, isomerization, and cyclization reactions to produce lycopene and its derivatives, such as β-carotene [15,16].

While the genetic framework is well-established, the final pigment profile and intensity in ripe fruit are profoundly modulated by environmental factors [17]. Light is a well-characterized regulator [18,19], influencing carotenoid accumulation through photoreceptor-mediated signaling [19,20] and the transcriptional activation of genes like *PSY1* [21,22]. In recent years, amid global warming and increasing frequency of extreme climatic events, temperature has also emerged as a crucial modulator of carotenoid metabolism across plant species, including tomato (*Solanum lycopersicum*) [23], sweet osmanthus (*Osmanthus fragrans* Lour.) [24], and citrus (*Citrus unshiu* Marc.) [25]. Deviations from optimal ripening temperatures can disrupt normal color development, often inhibit lycopene synthesis and lead to atypical yellow or orange phenotypes [17,25].

While extensive studies have examined light and temperature separately, their combined influence under natural or practical postharvest conditions remains poorly understood. The potential for synergistic or antagonistic interactions, along with the underlying molecular mechanisms, has yet to be elucidated. Furthermore, it is unclear whether pigment metabolism during ripening exhibits a defined temporal window of heightened sensitivity to these environmental cues. To address these gaps, we employed a semi-in-fruit experimental system to dissect the interactive effects of light and temperature on tomato coloration. This integrated approach aims to advance our understanding of environmental regulation in fruit ripening and support strategies for optimizing tomato quality in production and postharvest systems.

## 2. Results

### 2.1. Field Observations Reveal Temperature and Solar Radiation Associated Color Disorders

**Figure 1:**
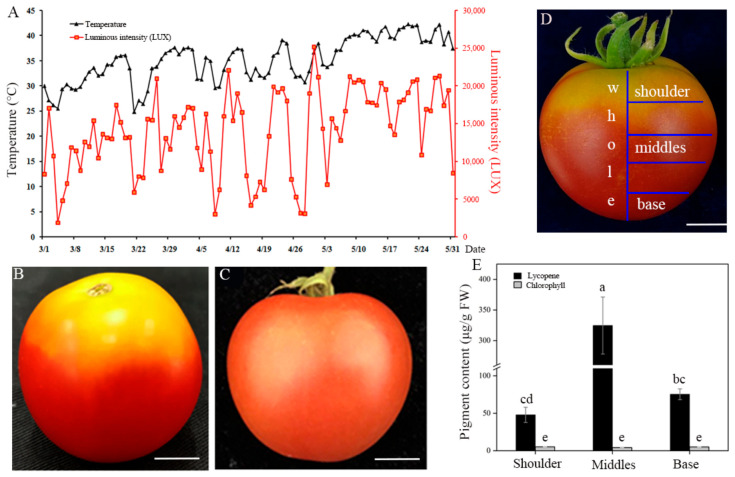
Comparison of the coloration of tomato fruit in different culture conditions. (**A**): Temporal and spatial variations in temperature and light intensity during the spring season in Guangzhou. (**B**): Phenotypic characteristics of tomato fruits cultivated during the spring season in Guangzhou. (**C**): Phenotypic characteristics of tomato fruits cultivated during the autumn season in Guangzhou. (**D**): Parts of the pericarp of uneven coloration tomato fruits. (**E**): The pigment content in three latitudinal sections of wild type ‘Ailsa Craig’ fruit. Different letters indicate that values are significantly different at *p* < 0.05.

Monitoring of environmental conditions in a parral greenhouse, situated on the campus of South China Agricultural University in Guangzhou City (23°7′ N, 113° E), from March to May recorded concurrent increases in maximum air temperature and solar radiation (Figure 1A). These sharp increases in light intensity were accompanied by drastic temperature fluctuations, coinciding with the onset of uneven coloration in ripening tomato fruits (Figure 1A). In contrast to uniformly red fruit harvested in autumn, tomatoes ripening during this period frequently developed yellow shoulders while the basal pericarps turned red (Figure 1B,C). Segmentation of these disordered fruits into shoulder, middle, and base regions (Figure 1D) and subsequent pigment analysis revealed significantly higher lycopene content in the middle and base compared to the shoulder, while chlorophyll was nearly undetectable in all sections (Figure 1E). This phenotype suggested a link between elevated temperature and solar radiation and the disruption of normal fruit coloration.

### 2.2. High Temperature Overrides Light to Inhibit Lycopene Accumulation

**Figure 2:**
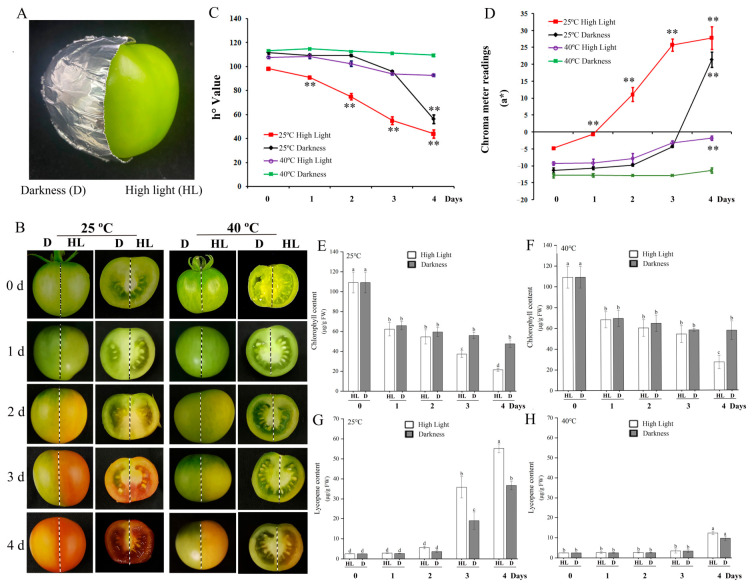
Analyses of the roles of light and temperature on tomato fruit color change. (**A**) Schematic diagram illustrating the experimental setup for tomato semi-fruit study. Each tomato fruit was subjected to two treatments: continuous intense light exposure on one half of the pericarp (HL treatment). In contrast, the other half was shaded with silver paper (dark treatment, D). Furthermore, these treatments were conducted at 25 °C and 40 °C; (**B**) Photographs showed the color change of the tomato fruit stored under different conditions (25 °C/HL, 25 °C/D, 40 °C/HL, and 40 °C/D); Changes of *h*^o^ (**C**) and a* (**D**) during storage at 25 °C/HL, 25 °C/D, 40 °C/HL, and 40 °C/D; contents of chlorophyll in different treatment under 25 °C (**E**) and 40 °C (**F**) in tomato pericarp during storage; Contents of lycopene in different treatment under 25 °C (**G**) and 40 °C (**H**) in tomato pericarp during storage. Data represent means ± the standard deviation of three biological repeats. Different letters indicate that values are significantly different at *p* < 0.05. Asterisks ** indicate significant differences *p* < 0.05 between 40 °C/D and other treatments at the same checkpoint.

To dissect the individual and interactive effects of light and temperature, we employed a semi-in-fruit system where one pericarp half received high light (HL) and the contralateral half was kept in darkness (D), under constant 25 °C or 40 °C (Figure 2A). Chlorophyll declined rapidly from day 1 under both 25 °C and 40 °C, with greater loss under HL at day 4 (Figure 2B,E,F). At 25 °C, HL-treated halves turned fully red by day 3, whereas D-treated halves remained greenish-yellow, though both achieved normal red coloration by the end of storage (Figure 2B,G). In contrast, at 40 °C, neither HL nor D treatment resulted in red pigmentation by day 4; HL-treated tissue turned yellow while D-treated tissue stayed light green (Figure 2B,H).

Colorimetric measurements confirmed these observations. At 25 °C, both treatments showed decreased hue angle (*h*^o^) and increased a* values, with changes significantly more pronounced under HL (Figure 2C,D). This correlated with faster chlorophyll degradation and higher lycopene accumulation in the 25 °C/HL group on day 3 (Figure 2E,G). In contrast, only minor shifts in *h*^o^ and a* occurred at 40 °C during storage (Figure 2C,D). Thus, high light accelerates ripening-associated pigmentation at optimal temperature (25 °C), but supra-optimal temperature (40 °C) completely inhibits lycopene biosynthesis irrespective of light.

### 2.3. Transcriptional Suppression of PSY1 and GGPS2 Underlies Heat-Induced Lycopene Inhibition

**Figure 3:**
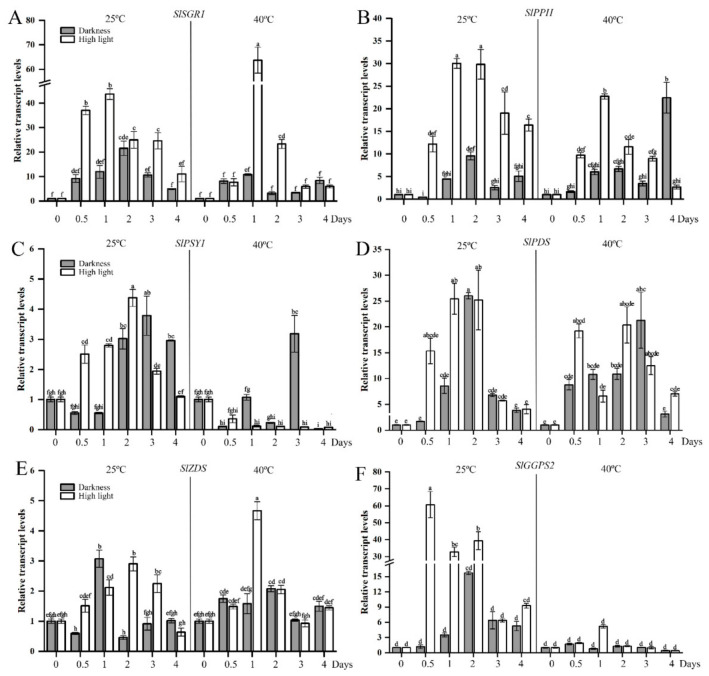
The chlorophyll degradation and carotenoid biosynthetic-related genes in tomato pericarp. (**A**–**F**): Changes in *SlSGR1*, *SlPPH*, *SlPSY1*, *SlPDS*, *SlZDS*, and *SlGGPS2* transcription levels in tomato pericarp following 25 °C/HL, 25 °C/D, 40 °C/HL, and 40 °C/D treatments. Data represent means ± the standard deviation of three biological repeats. Different letters indicate that values are significantly different at *p* < 0.05.

Expression analysis of chlorophyll degradation genes (*SlSGR1*, *SlPPH*) revealed rapid upregulation during storage under both temperatures, which peaked at day 1 or day 2 (Figure 3A,B). Notably, expression levels were consistently higher under HL than in darkness at both temperatures, aligning with the observed accelerated chlorophyll loss under light.

The key lycopene biosynthesis genes, including *SlPSY1*, *SlPDS*, *SlZDS*, and *SlGGPS2*, were strongly induced and exhibited high expression levels during storage at 25 °C, with HL further enhancing their transcript levels compared to darkness (Figure 3C–F). In contrast, the expression of *SlPSY1* and *SlGGPS2* was severely suppressed to trace levels in both light and dark conditions at 40 °C, while *SlPDS* and *SlZDS* showed no significant induction (Figure 3C–F). These results indicate that high temperature primarily disrupts carotenoid biosynthesis by repressing the transcription of critical early genes, particularly *SlPSY1* and *SlGGPS2*.

### 2.4. Temperature-Dependent Shift in Carotenoid Profiles

**Figure 4:**
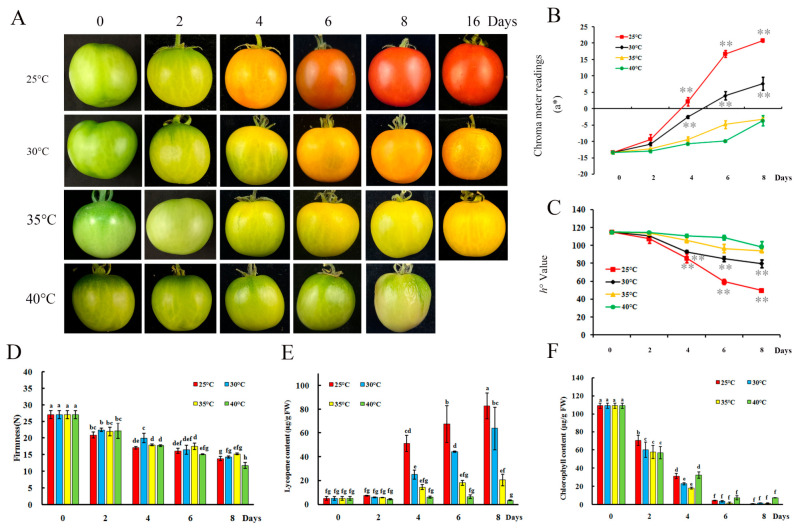
Changes in fruit pericarp color of tomato during ripening at different temperatures. (**A**): Appearance of tomato fruit stored at 25 °C, 30 °C, 35 °C and 40 °C; changes of a* (**B**) and *h*^o^ (**C**) during storage at 25 °C, 30 °C, 35 °C and 40 °C; (**D**): Change in the firmness of tomato fruit during storage at 25 °C, 30 °C, 35 °C and 40 °C. Contents of lycopene (**E**) and chlorophyll (**F**) in tomato pericarp were detected during storage after ethylene treatment at 25 °C, 30 °C, 35 °C, and 40 °C. Data represent means ± the standard deviation of three biological repeats. Different letters indicate that values are significantly different at *p* < 0.05. Asterisks ** indicate significant differences *p* < 0.05 between 40 °C and other temperature at the same checkpoint.

To identify the optimal temperature for carotenoid biosynthesis in ripening tomato fruit, mature green (MG) tomatoes were stored under controlled dark conditions at 25 °C, 30 °C, 35 °C, and 40 °C, respectively (Figure 4A). Fruits at 25 °C, 30 °C, and 35 °C transitioned from green to red, orange, or yellow over 8 days, while those at 40 °C remained green and later decayed. Firmness decreased across all temperatures, most rapidly at 40 °C (Figure 4D). Colorimetry showed the greatest increase in a* and decrease in *h*^o^ at 25 °C, with moderated changes at 30 °C and 35 °C, and minimal variation at 40 °C (Figure 4B,C). Lycopene accumulation, as determined by spectrophotometry, reached its maximum at 25 °C and declined sharply at temperatures above 30 °C, becoming undetectable at 40 °C (Figure 4E). HPLC analysis further revealed a temperature-dependent shift in carotenoid composition (Table 1): lycopene (145.73 μg/g) was predominant only at 25 °C. At 30 °C and 35 °C, α-carotene (254.89 and 46.01 μg/g), β-carotene (55.15 and 75.58 μg/g), and lutein (78.83 and 117.46 μg/g) became the major pigments. At 40 °C, lutein (119.00 μg/g) and a trace of violaxanthin (8.63 μg/g) were present. Chlorophyll content decreased across all treatments, becoming undetectable under 25 °C to 35 °C at the end of storage, while the 40 °C group retained approximately 9% of its original content, aligning with the green ripening phenotype (Figure 4F).

### 2.5. Elevated Temperature Compromises Nutritional Quality

Nutritional quality of fruit at day 8 analysis showed a clear negative impact of increasing temperature. Antioxidant activity, assessed by DPPH and hydroxyl radical scavenging assays, were highest in fruits stored at 25 °C and 30 °C, significantly lower at 35 °C, and lowest at 40 °C (Figure S1A,B). Similarly, vitamin C content declined progressively with rising temperature, with the maximum retention at 25 °C and the minimum at 40 °C (Figure S1C). However, there was no significant difference in soluble solids content among the different temperature treatments on day 8 (Figure S1D). These results demonstrate that postharvest temperatures above 30 °C accelerate the loss of key nutritional attributes.

The expression patterns of *SlPSY1*, *SlGGPS2*, *SlACO1*, and *SlACS4* were characterized on day 4 (Figure S2A–D). The transcript levels of all four genes were highest in fruits stored at 25 °C and 30 °C, significantly lower at 35 °C, and lowest at 40 °C. Overall, as the temperature increased above 30 °C, transcript levels decreased significantly, showing a clear temperature-dependent inhibition, with the most severe suppression occurring at 40 °C.

### 2.6. A Critical Post-Ethylene Window Determines Temperature-Mediated Color Fate

**Figure 5:**
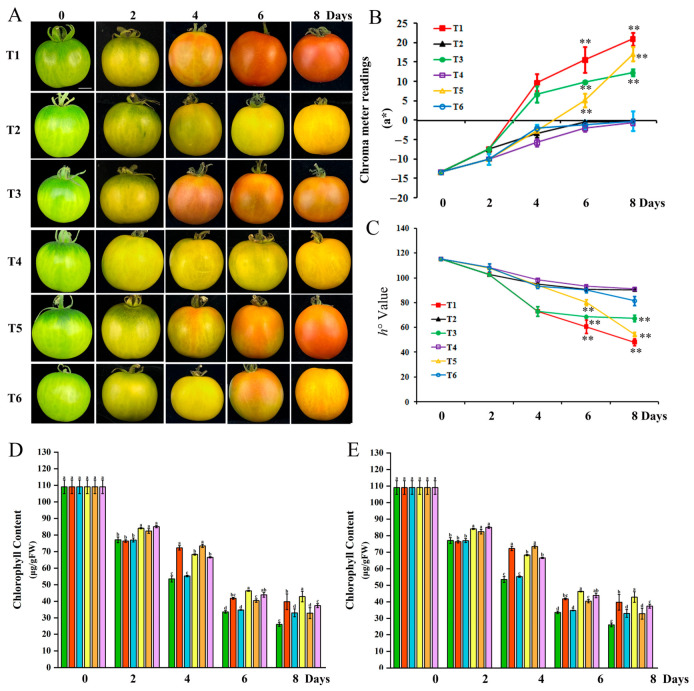
Changes in pericarp color of tomato during ripening held continuously or at 25 °C or 35 °C. (**A**): The tomato fruit after 8 days of ripening following T1–T6 temperature schedules. The ripening initiation and treatments are described in Table 1. (**B**): Changes in a* of tomato fruits following T1–T6 treatments during ripening held constant at 25 °C or 35 °C. (**C**): Changes in *h*^o^ of tomato fruits following T1–T6 treatments during ripening held constant at 25 °C or 35 °C; (**D**): Contents of chlorophyll in T1–T6 fruit during storage; (**E**): Contents of lycopene in T1–T6 fruit during storage. The values presented are means of three measurements taken at three individual fruits of the same treatment. Error bars indicate the SD of the values. The different letters demonstrated significant differences at the same checkpoint via Tukey’s multiple comparison test (*p* < 0.05). Asterisks ** indicate significant differences *p* < 0.05 between T4 and other treatments at the same checkpoint.

To test temporal sensitivity, green mature fruits after ethylene treatment were subjected to temperature-switch protocols during an 8-day ripening period in darkness (Table 1). Fruits kept constantly at 25 °C (T1) or switched from 25 °C to 35 °C on day 4 (T3) developed full red coloration (Figure 5A–C). Similarly, fruits switched from 35 °C to 25 °C on day 2 (T5) also turned red. The *h*^o^ and a* values showed marked reddening in fruits T1, T3 and T5 (Figure 5A–C). In contrast, fruits kept constantly at 35 °C (T4), shifted from 25 °C to 35 °C on day 2 (T2), or moved from 35 °C to 25 °C on day 4 (T6) ripened to yellow or orange hues (Figure 5A). Consistently, *h*^o^ and a* values in T2, T4, and T6 remained relatively stable throughout storage (Figure 5B,C).

Chlorophyll content declined progressively in all treatment groups during storage, with no significant differences detected among treatments at any individual time point (Figure 5D). Fruits from T1, T3, and T5 accumulated substantial lycopene during ripening, reaching the highest level in T1; however, lycopene remained barely detectable in T2, T4, and T6 (Figure 5E). Together, these results demonstrate that a sustained period at 25 °C within this early window is essential for normal red coloration, while high-temperature exposure during this phase irreversibly shifts carotenoid metabolism toward the accumulation of yellow and orange pigments.

### 2.7. SlPSY1 and SlGGPS2 Expression Mirrors Stage-Specific Temperature Sensitivity

**Figure 6:**
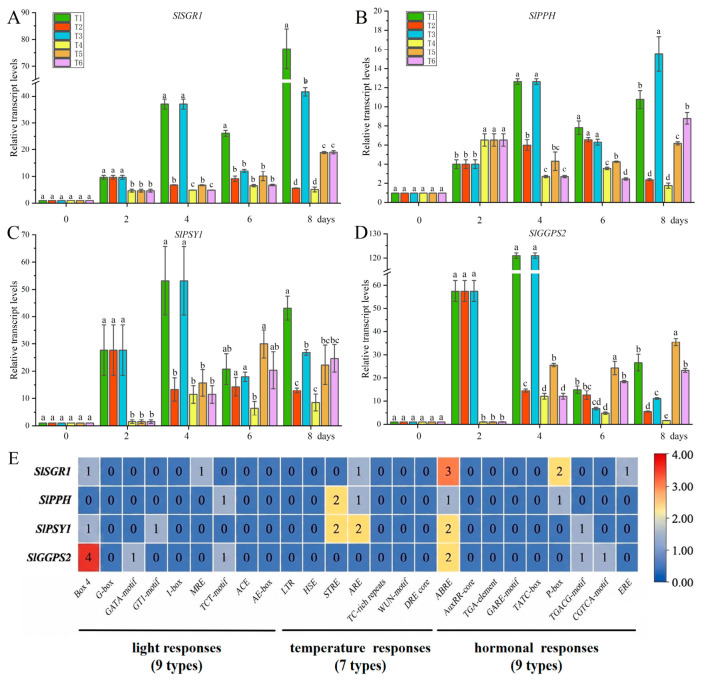
The expression levels of chlorophyll degradation and carotenoid biosynthetic-related genes in tomato during ripening held continuously or at 25 °C or 35 °C. (**A**–**D**): Quantitative real-time PCR of *SlSGR1*, *SlPPH*, *SlPSY1*, and *SlGGPS2* transcription levels in tomato pericarp following T1–T6 treatments during ripening held at constant at 25 °C or 35 °C. *SlSGR1*, *SlPPH*, *SlPSY1*, and *SlGGPS2* transcript levels in T1–T6 treatments during ripening at day 0 were set to 1. (**E**): *Cis*-acting element analysis of the promoter region of *SlSGR1*, *SlPPH*, *SlPSY1*, and *SlGGPS2*. The *cis*-elements located in the 2000 bp promoter sequence (upstream of the start codon) of these genes were analyzed via the *PlantCARE* website. The number of corresponding *cis*-acting elements was used for the heatmap construction. The different lowercase letters indicate significant differences among the plants on the same day according to Tukey’s multiple comparison test (*p* < 0.05). Data represent means ± the standard deviation of three biological repeats.

The expression of chlorophyll degradation (*SlSGR1*, *SlPPH*) and lycopene biosynthesis (*SlPSY1*, *SlGGPS2*) genes closely tracked the temperature-switch phenotypes (Figure 6). A fruit maintained at 25 °C (T1), the expression of *SlSGR1*, *SlPPH*, *SlPSY1* and *SlGGPS2* gradually increased upon the initiation of ripening, reaching peak levels after day 4. The fruit in T2 caused a marked suppression of *SlSGR1* and *SlPPH* from day 4, and a sharp decline in *SlPSY1* and *SlGGPS2* after the switch (Figure 6A–D). Similarly, a decrease in the expression of *SlPSY1* and *SlGGPS2* was noted in T3 fruit on days 6 and 8 following their transfer from 25 °C to 35 °C while the expression of *SlSGR1* and *SlPPH* was not affected. Fruit placed at a constant temperature of 35 °C (T4) exhibited an initial increase in the expression of these biosynthetic genes from day 1, followed by a sustained relatively low expression level during storage. Conversely, transferring fruits from 35 °C to 25 °C (T5, day 2) triggered a recovery in the expression of *SlPSY1* and *SlGGPS2* from day 4 onwards, coinciding with successful red color development. A delayed transfer (T6, day 4) resulted in only partial gene expression recovery by day 6, insufficient to rescue the lycopene pathway.

Using the PlantCare database [26], the distribution of cis-elements in the upstream 2 kb promoter regions of four tomato genes, *SlSGR1*, *SIPPH*, *SIPSY1*, and *SIGGPS2*, was analyzed (Figure 6E). In this study, we found that 2, 1, 2, and 5 light-responsive cis-elements were present in the promoters of *SlSGR1*, *SIPPH*, *SIPSY*, and *SIGGPS*, respectively. In addition, 1, 3, 4, and 0 temperature-responsive cis-elements were identified in the promoters of *SlSGR1*, *SIPPH*, *SIPSY*, and *SIGGPS*, respectively. Collectively, these expression and promoter analyses define a critical window (days 2–4) during which the transcription of key pigmentation genes is rapidly and reversibly modulated by temperature, although the temperature sensitivity of *SlGGPS2* appears to be independent of canonical temperature-responsive elements in its own promoter.

## 3. Discussion

The coloration of tomato fruits is a pivotal indicator of its palatability and has garnered significant attention from researchers aiming to characterize and quantify this attribute [27]. The ultimate brightness of tomato color is influenced by a complex interplay of genetic predispositions and environmental conditions [17]. In this study, we systematically investigated the combined effects of light and temperature on the metabolism of chlorophylls and carotenoids in tomato fruit.

Previous studies have confirmed that light and temperature each individually influence lycopene synthesis in tomato fruits during ripening [19,20,28]. In general, mild to moderate light stress can benefit fruit pigment accumulation [17]. Our findings show that tomato fruit pericarps transition from green to red under both high light (HL) and dark conditions when maintained at a constant 25 °C. However, under 25 °C/HL condition, chlorophyll breakdown and lycopene accumulation was more rapid and pronounced than under 25 °C/D condition (Figure 2). This accelerated process under 25 °C/HL can be attributed to the upregulated expression of genes involved in chlorophyll degradation and carotenoid synthesis, such as *SlSGR1*, *SlPPH*, *SlPSY1*, and *SlGGPS2* (Figure 2B and Figure 3). Similar results were reported by [20,29], who found that red light increased lycopene and β-carotene levels in tomato fruits compared to darkness, associated with the upregulation of *PSY1* and other carotenoid biosynthesis genes. The effect of temperature, especially high temperature, on carotenoid synthesis in fruits is complex. High temperature can either promote or inhibit carotenoid accumulation, depending on the crop species. For instance, hot chili peppers kept at 30 °C exhibited a more intense red color and higher carotenoid content [30]. In banana pulp, elevated temperature also increased carotenoid levels, presumably via upregulation of *MaDXR1*, *MaPDS1*, *MaZDS1*, and *MaLCYE* [31]. In this study, tomato held at 30 °C decreased not only the contents of chlorophyll, but also the synthesis of lycopene and β-carotene. Notably, lycopene synthesis was completely suppressed when the temperature exceeded 40 °C, irrespective of light conditions (Figure 2A and Figure 4A). This suppression under high temperature is consistent with previous reports in tomato [32] and other species [24,33], which indicated that temperature thus overrides light signals at the transcriptional level.

It is worth noting that heat stress typically accelerates chlorophyll degradation in leaves from creeping bentgrass species and other plants [34,35]. Conversely, tomato fruits stored at 40 °C in darkness remained green at the ripe stage (a “green-ripe” phenotype). The observed pericarp coloration patterns were consistent with the downregulation of genes involved in chlorophyll degradation and lycopene synthesis (Figure 4A–C,F). This phenomenon echoes similar trends observed in banana fruit, where temperatures exceeding 30 °C facilitate normal softening and ripening but suppress chlorophyll degradation in the peel, leading to a stay-green phenotype caused by incomplete chlorophyll breakdown regulated by chlorophyll catabolic gene [36,37,38]. This inconsistency in leaves and fruits may be attributed to the distinct physiological responses of different plant tissues to high-temperature stress.

Considering that lycopene synthesis in tomatoes requires ethylene [39], many previous studies have shown that ethylene treatment at 20–24 °C promotes ripening and color uniformity [40]. Our study demonstrates that when the temperature reaches or exceeds 30 °C, ethylene treatment becomes ineffective in accelerating color development (Figure 4A). This ineffectiveness correlates strongly with the suppressed expression of the ethylene biosynthesis genes *SlACO1* and *SlACS4* above 30 °C (Figure S2C,D). Similar results have been reported, showing that ethylene treatment at 40 °C resulted in the lowest ACO activity compared to other temperatures [41]. However, reduced ethylene biosynthesis alone does not fully account for color change. Yang et al. [42] found that even when exogenous ethylene was applied to mature-green tomatoes stored at 30 °C or 37 °C, the suppression of color development was not overcome. Taken together, our results suggest that the temperature effect on pigmentation may be mediated through a dual mechanism. Directly, the promoters of *SlPSY1*, *SlPPH*, and *SlSGR1* contain canonical temperature-responsive elements (four, three, and one copy(s), respectively), which may allow rapid transcriptional responses upon temperature shift (Figure 6C,E). Indirectly, high temperature appears to suppress ethylene biosynthesis (*SlACO1*/*SlACS4*) and signaling, thereby potentially reducing ethylene-driven expression of both chlorophyll-degradation and lycopene-biosynthesis genes.

The timing of heat exposure is critical. In an 8-day ripening schedule, notable red fruits were observed in the T1, T3, and T5 treatments. Conversely, fruits in T2, T4, and T6 exhibited yellow or orange-like ripening phenotype (Figure 5). The results showed that the first 4 days, or precisely the 3rd to 4th day was most critical for the high-temperature effect on the color change in tomato fruit. During this period, tomato fruits typically reach their respiratory peak [43,44], and Chl degradation precedes carotenoid biosynthesis [44,45]. Disruption of this temporal coordination by high temperature can irreversibly compromise normal ripening, although partial recovery is possible if the heat stress is relieved before the window closes, such as seen in the T3 and T5 treatments. The molecular basis for this temperature sensitivity is evident in the expression patterns of key genes. The expression of several Chl degradation and lycopene synthesis genes, namely *SlSGR*, *SlPPH*, *SlPSY1*, and *SlGGPS2*, was highly sensitive to temperature changes in this study (Figure 6). A significant reduction or increase in *SlSGR*, *SlPPH*, *SlPSY1*, and *SlGGPS2* was detected 2 days after a shift from 25 °C to 35 °C or 35 °C to 25 °C (Figure 6). The results in other fruit also confirmed that *SGR1* [39,46] and *PSY1* [24,47] were sensitive to high temperature. Promoter cis-element analysis (Figure 6E) revealed that *SlPSY1* contains four temperature-responsive elements, consistent with its strong downregulation under high temperature. All four promoters harbor light-responsive elements (two, one, two, and five copies, respectively), explaining why light promotes pigmentation at permissive temperatures. Mechanistically, our data suggest that high temperature (≥30 °C) suppresses lycopene accumulation, with direct repression of *SlPSY1* through its temperature-responsive promoter elements playing a potential role.

In conclusion, high temperature (≥30 °C) not only overrides light-induced lycopene synthesis but also compromises fruit quality by reducing antioxidant activity and vitamin C content; however, the precise molecular mechanism remains to be determined. These findings highlight the critical need to avoid heat stress during tomato cultivation, particularly at the mature green stage, in order to preserve normal ripening and nutritional quality.

## 4. Materials and Methods

### 4.1. Plant Material and Treatments

Tomato (*Solanum lycopersicum* cv Ailsa Craig) were grown in pots (330 × 290 mm) with sterilized soil (Jiffy7^®^, Jiffy Products International B.V., Zwijndrecht, The Netherlands) in a growth chamber under the following conditions: 16/8 h light/dark at 25/18 ± 2 °C (250 µmol m^−2^ s^−1^) and 65 ± 5% relative humidity. The plants were fed with fertilizer (N-P_2_O_5_-K_2_O:15-6-8) every two weeks. The fruits tagged at anthesis (DPA, days post anthesis) were collected at 40 DPA.

The selected pre-climacteric tomato fruits were first surface sanitized by dipping in a 1% hypochlorite solution for 3 min and then immersed in 0.05% Ethephon for 1 min to initiate ripening. For each temperature treatment, three independent polyethylene bags (0.01 mm thickness, with small perforations for air exchange) each containing 20 fruits were used as three biological replicates. At each daily sampling time point, one fruit was taken randomly from each bag, yielding three biological replicates per time point. From each sampled fruit, color, firmness, and pericarp tissue were measured or collected. The pericarp tissues were immediately frozen in liquid N_2_ and stored at −80 °C until further use. Subsequent experiments were performed using a similar sampling strategy.

For temperature-switch experiment, six temperature treatments (T1–T6) were applied as indicated in Table 2. Tomato fruits were equally distributed into three replicate bags, each containing 20 fruits. Tomato fruits were treated with 0.05% Ethephon for 1 min to initiate ripening and kept for another 8 days continuously or at 25 °C or 35 °C. Treatment 1 (T1): fruits were held at 25 °C continuously for 8 days; treatment 2 (T2): fruits were moved to 35 °C after ripening at 25 °C for 2 days; treatment 3 (T3): fruits were transferred to 35 °C after ripening at 25 °C for 4 days; treatment 4 (T4): fruits were held at 35 °C continuously; treatment 5 (T5): fruits moved to 25 °C after ripened at 35 °C for 2 days; treatment 6 (T6): fruits were moved to 25 °C after ripened 35 °C for 4 days.

Semi-fruit experimental system: All experiments were conducted inside a temperature-controlled growth chamber (incubator) set at 25 °C or 40 °C. Half of the mature green tomato fruit was exposed to continuous intense light (680 µmol m^−2^ s^−1^, high light treatment), and the contralateral half was shaded with silver paper (dark treatment). To exclude the possibility of localized heating, a thermometer was placed at the same height as the fruit under identical light conditions inside the incubator. The tomato pericarp from the HL and darkness treatment were collected at 0.5, 1, 2, 3, and 4 days, respectively. Samples were immediately frozen in liquid nitrogen and stored at −80 °C.

### 4.2. Color Measurement

Three fruits per treatment were measured for pericarp color. An NR-3000 color spectrophotometer (NIPPON DENSHOKU, Tokyo, Japan) was used to test three positions around the equator of each fruit. CIE color space coordinates were recorded, including hue angle (*h*^o^) and a*.

### 4.3. Determination of Firmness

Firmness was measured using a universal traction–compression machine (Instron 5542, Instron Corp., Norwood, MA, USA) with computerized data acquisition equipped with a 4 mm diameter flat plunger. Measurements were made on each fruit of three fruits per treatment at three different locations from bottom to top. The firmness was recorded as the maximum peak force in Newton (N) achieved during compression and extrusion.

### 4.4. Pigment Extraction and Quantification

For spectrophotometric determination of chlorophyll concentrations, frozen peel pieces (1 g) were ground in liquid nitrogen and placed into 10 mL cold aqueous acetone (80%, *v*/*v*) containing 1 mM KOH overnight in the dark at 4 °C. After centrifugation at 10,000 g for 10 min, the residue was re-extracted with cold aqueous acetone until it became colorless. All the supernatants were combined and brought to 20 mL. The spectrophotometer measured the absorbance of the Chl supernatant at 663 nm and 645 nm. The Chl concentration per fresh weight of the peel pieces was calculated as described [9].

One gram of frozen peel pieces was ground in liquid nitrogen, subsequently extracted in hexane/acetone/ethanol (50:25:25), and afterward centrifuged at 3000× *g* for 5 min at 4 °C. Immediately after extraction, the absorbance of lycopene was measured at 472 nm using hexane as a reference. The total lycopene concentration was calculated using the Beer–Lambert law [47]:

*C* = (*A* × 10 × 10^4^)/(*E*_1cm_ × 10^18^ × *l*) (1)

with *C* the total lycopene concentration (μg/mL), A the absorbance at 472 nm, *E*_1cm_ × 10^18^ the extinction coefficient of lycopene in hexane [=3450 (g/100 mL)^−1^ cm^−1^] and *l* the path length (=1 cm).

### 4.5. HPLC Analysis of Carotenoids

Extraction of carotenoid was carried out by the method of Baroli et al. [48] with modifications. Pigments were extracted by vortexing in 500 L of acetone at maximum speed for 15 min, and afterward centrifuged at 12,000× *g* for 15 min at 4 °C; the extracts were filtered through a 0.45 m nylon filter. Twenty microliters of each extract were separated by HPLC on a Waters Spherisorb S5 ODS2 4.6–250 mm cartridge column (Milford, MA, USA). Pigments were eluted at a flow rate of 1.2 mL/min with a linear gradient from 100% solvent A (acetonitrile: methanol: 0.1 M Tris-HCl, pH 8.0 [84:2:14]) to 100% solvent B (methanol:ethyl acetate [68:32]) for 15 min, followed by 3 min of solvent B. Pigments were detected at 445 nm with 550 nm as the reference wavelength. Analytes were identified and calculated by comparison of their retention time with those of authentic standards (lycopene, β-carotene α-carotene, violaxanthin and lutein). Results were represented as mg per 100 g dry mass.

### 4.6. RNA Isolation

Total RNA was isolated from 100 mg of tomato leaves or fruit pericarps with a TRIzol reagent (Invitrogen, Carlsbad, CA, USA), and cDNA was synthesized with a Prime-Script cDNA Synthesis Kit (TaKaRa, Shiga, Japan) following the manufacturer’s manuals.

### 4.7. Gene Expression Analysis

The expression levels of genes were quantified by qRT-PCR using gene-specific primers (listed in Table S1 [49,50,51]) with ChamQ Universal SYBR qPCR Master Mix (Vazyme Biotech Co., Ltd. Nanjing, China) on a CFX Connect TM Real-Time System (Bio-Rad, Hercules, CA, USA). A PCR reaction mix contained 10 µL of ChamQ Universal SYBR qPCR Master Mix (Vazyme Biotech Co., Ltd., Nanjing, China), 125 nM of each forward and reverse primer, 2.0 µL of cDNA template, and 7.5 µL of RNase-free water. Data analysis was calculated using the 2^−∆Ct^ or 2^−∆∆Ct^ methods [52]. All experiments were measured in three biological replicates.

### 4.8. Antioxidant Activity Determination by Chemical-Based Assays

Freeze-dried tomato pericarp was ground into a fine powder in liquid nitrogen, then the powder (100 mg) was dissolved in 1 mL of HCl-methanol solution and extracted with shaking at room temperature for 2 h. Subsequently, the samples were centrifuged at 1000 g for 90 s. The supernatant was collected and stored at −20 °C.

#### 4.8.1. Hydroxyl Radical Scavenging Activity Assay

The scavenging activity was calculated according to the method described by Cai et al. [53]. In a tube, 2.0 mL of 6.0 mmol/L ferrous sulfate solution was added first, followed by 2 mL of sample solution, and the mixture was gently shaken. Subsequently, 2.0 mL of 0.1% (*w*/*v*) H_2_O_2_ was added, mixed gently, and allowed to stand for 15 min. Then, 2.0 mL of 6.0 M salicylic acid solution was added, gently shaken, and left to stand for 30 min. Finally, the mixture was diluted to the mark with distilled water, incubated in a water bath at 50 °C for 30 min, and the absorbance was measured at 510 nm against a distilled water blank. The hydroxyl radical scavenging rate was calculated using the following formula, where AS is the absorbance of the sample, A1 is the absorbance of the control solution containing 1,10-phenanthroline, FeSO_4_, and H_2_O_2_, and A0 is the absorbance of the blank solution containing 1,10-phenanthroline and FeSO4.

Hydroxyl radical scavenging activity(%) = 0.5 × (AS − A1)/(A0 − A1) × 100,(2)

#### 4.8.2. DPPH Radical Scavenging Activity Assay

The DPPH radical-scavenging activity of tomato extract was determined by a UV–visible plate reader according to the previous report [54]. A 30 µL aliquot of the sample solution was mixed with 585 µL of 50 µM DPPH and incubated in the dark for 6 h. Wells with 30 μL sample solution and 585 μL methanol were set as blanks to avoid the interference of the sample itself. The DPPH radical-scavenging activity was expressed as μmol Trolox equivalent per gram of tomato in dry weight (μmol TE/g DW).

### 4.9. Ascorbic Acid Analysis

The ascorbic acid content was quantified using the 2,6-dichlorophenolindophenol (DCPIP) titration method as described by Song et al. [20]. Briefly, 0.2 g of freeze-dried sample was homogenized in 5 mL of 2% oxalic acid solution and subsequently filtered. A 1 mL aliquot of the filtrate was diluted with 5 mL of oxalic acid solution and then titrated with a 0.01% DCPIP solution. The endpoint was determined by the appearance of a faint, persistent beige color lasting 30 s. Ascorbic acid concentration was calculated based on the volume of DCPIP solution consumed and expressed as mg per gram of dry mass.

### 4.10. Total Soluble Solids Content

Three independent fruits were used for total soluble solids (TSS) measurement. Each fruit was homogenized using a blender, and the homogenate was filtered through cheesecloth. A drop of the filtrate was then placed onto the glass surface of a handheld refractometer (ATAGO, Tokyo, Japan), and the TSS content (ºBrix) was recorded. The refractometer was zeroed with ddH_2_O before each measurement.

### 4.11. In Silico Analysis of Cis-Elements in Promoters of Genes

The promoter sequences (2 kb of the 5′ upstream region of the start codon) of the *SlPSY1*, *SlGGPS2*, *SlSGR1* and *SlPPH* genes were extracted from the International Tomato Genome Sequencing Consortium (SGN; http://www.solgenomics.net) database (version ITAG 2.4). The data was accessed on 5 May 2026. The PlantCARE relational database [26] was used for plant cis-element searches in the promoter of these genes.

### 4.12. Statistical Analysis

The experiments were arranged in a completely randomized design, and each treatment comprised three replicates. For time-course data involving two factors (Figure 1, Figure 2 and Figure 4), two-way repeated-measures ANOVA was used; for the three-factor design in Figure 3, three-way repeated-measures ANOVA was applied, including all interaction terms. For cross-sectional comparisons at each time point (Figure 5 and Figure 6), one-way ANOVA followed by Tukey’s HSD test was performed. Pearson correlation coefficients were calculated, and a two-tailed test was used to determine significance at the 1% and 5% levels. Statistical analyses were performed with SPSS19.0 (SPSS, Chicago, IL, USA).

## 5. Conclusions

Based on the findings of this study, we propose a model for the environmental regulation of tomato fruit coloration. Light and temperature do not act as independent modulators but rather operate within a hierarchical framework, wherein temperature exerts dominant control during a narrowly window-time. From a practical standpoint, these findings suggest that targeted temperature management during this narrow window, rather than throughout the entire ripening period, may be sufficient to ensure desirable fruit color and nutritional quality.

## Figures and Tables

**Table 1:** Carotenoid profiles of tomato fruit stored at different temperatures for 8 days.

Treatment	Lycopene (μg/g)	α-Carotene (μg/g)	β-Carotene (μg/g)	Lutein (μg/g)	Violaxanthin (μg/g)
25 °C	145.73 ± 14.38	ND	2.72 ± 17.15	32.58± 41.35	ND
30 °C	ND	254.89± 61.45	55.15 ± 8.46	78.83 ± 17.04	ND
35 °C	ND	46.01 ± 0.60	75.58 ± 8.62	117.46 ± 22.37	ND
40 °C	ND	ND	23.85 ± 3.35	119.00 ± 30.83	8.63 ± 0.409

**Table 2:** The eight-day temperature changing regimes applied for tomato fruit ripening.

Treatments	Day 1	Day 2	Day 3	Day 4	Day 5	Day 6	Day 7	Day 8
T1	25 °C	25 °C	25 °C	25 °C	25 °C	25 °C	25 °C	25 °C
T2	25 °C	25 °C	35 °C	35 °C	35 °C	35 °C	35 °C	35 °C
T3	25 °C	25 °C	25 °C	25 °C	35 °C	35 °C	35 °C	35 °C
T4	35 °C	35 °C	35 °C	35 °C	35 °C	35 °C	35 °C	35 °C
T5	35 °C	35 °C	25 °C	25 °C	25 °C	25 °C	25 °C	25 °C
T6	35 °C	35 °C	35 °C	35 °C	25 °C	25 °C	25 °C	25 °C

## Data Availability

The original contributions presented in this study are included in the article/Supplementary Materials. Further inquiries can be directed to the corresponding author.

## Supplementary Materials

The following supporting information can be downloaded at: https://www.mdpi.com/article/10.3390/ijms27114690/s1.
