# Supplementary material for: Temperature Dominates Light in Regulating Lycopene During a Critical Period in Postharvest Tomato Fruit
Source: Int J Mol Sci. 2026 May 22;27(11):4690. doi: 10.3390/ijms27114690 (PMC13256695; doi:10.3390/ijms27114690)
Supplement: Supplementary file 1 [file ijms-27-04690-s001.zip › ijms-4281287-supplementary.pdf]

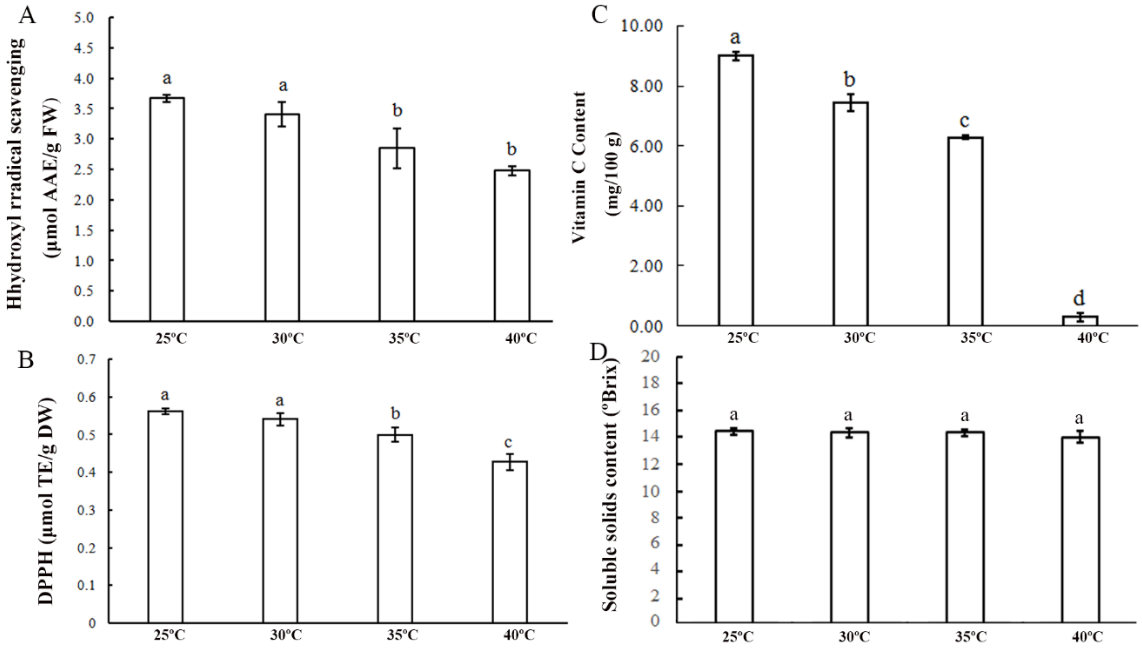


Figure S1: Effects of different temperature on Fruit Nutritional Quality. Hydroxyl radical scavenging assays (A), DPPH (B), ascorbic acid (C), and soluble solids content(D). The different lowercase letters indicate significant differences among the plants on the same day according to Tukey's multiple comparison test ($p < 0.05$). Data represent means ± the standard deviation of three biological repeats.

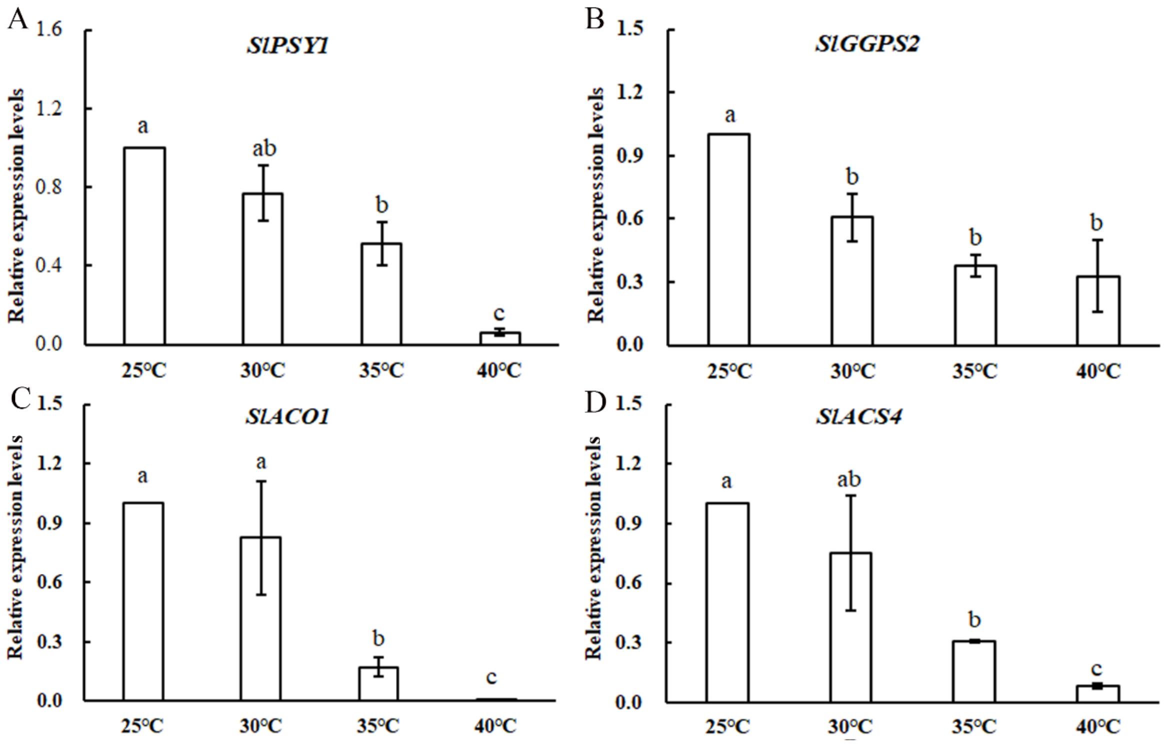


Figure S2: The carotenoid and ethylene biosynthetic-related genes in tomato pericarp (A–D): Changes in *SlPSY1*, *SlGGPS2*, *SlACO1*, and *SlACS4* transcription levels in tomato pericarp following 25 °C, 30 °C, 35 °C, and 40 °C treatments at day 4. The different lowercase letters indicate significant differences among the plants on the same day according to Tukey's multiple comparison test ($p < 0.05$). Data represent means ± the standard deviation of three biological repeats.

Supplemental Table S1 The primers used in this study

| Gene symbol | Primer name | Sequence (5′→3′) |
| --- | --- | --- |
| *SlEF-1α* [49] | Actin-F | AGATGGTCAGACCCGTGAAC |
| | Actin-R | TGGAGTACTTGGGGGTGGTA |
| *SlACO1* [50] | ACO1-F | GCCAAAGAGCCAAGATTTGA |
| | ACO1-R | TTTTTAATTGAATTGGGATCTAAGC |
| *SlACS4* [51] | ACS4-F | CTCCTCAAATGGGGAGTACG |
| | ACS4-F | TTTTGTTTGCTCGCACTACG |
| *SlSGR1* | SGR1-F | AAAATGGGACCATCCAACAA |
| | SGR1-R | GCTGCTTCCACAAACCCTAT |
| *SlPPH* | PPH-F | TATGGAGGGAGCAAGTACGC |
| | PPH-R | TGGAGGGCAGAGGAAAAGTAC |
| *SlPSY1* | PSY1-F | CGATGGTGCTTTGTCCGATAC |
| | PSY1-R | CTCATCAACCCAACCGTACC |
| *SlGGPS2* | GGPS2-F | GGGATTGGAAAAGGCTAAGG |
| | GGPS2-R | AGCAATCAATGGAGCAGCTT |
| *SlZDS* | ZDS-F | ACCGTACAACTACGCTACAATGG |
| | ZDS-R | CATCTGGCGTATAGAGGAGATTG |
| *SlPDS* | PDS-F | CGTTCCGTGCTTCTCCGC |
| | PDS -R | CTAGAACATCCCTTGCCTCCAG |
